# Temporomandibular Joint Injections and Lavage: An Overview of Reviews

**DOI:** 10.3390/jcm13102855

**Published:** 2024-05-12

**Authors:** Natalia Turosz, Kamila Chęcińska, Maciej Chęciński, Karolina Lubecka, Filip Bliźniak, Dariusz Chlubek, Tomasz Olszowski, Maciej Sikora

**Affiliations:** 1 Department of Maxillofacial Surgery, Hospital of the Ministry of Interior, Wojska Polskiego 51, 25-375 Kielce, Poland; natalia.turosz@gmail.com (N.T.); sikora-maciej@wp.pl (M.S.); 2 Department of Glass Technology and Amorphous Coatings, Faculty of Materials Science and Ceramics, AGH University of Science and Technology, Mickiewicza 30, 30-059 Cracow, Poland; checinska@agh.edu.pl; 3 Department of Oral Surgery, Preventive Medicine Center, Komorowskiego 12, 30-106 Cracow, Poland; maciej@checinscy.pl (M.C.); lubeckarolina@gmail.com (K.L.); fblizniak@gmail.com (F.B.); 4 Department of Biochemistry and Medical Chemistry, Pomeranian Medical University, Powstańców Wielkopolskich 72, 70-111 Szczecin, Poland; 5 Department of Hygiene and Epidemiology, Pomeranian Medical University, Powstańców Wielkopolskich 72, 70-111 Szczecin, Poland; tomasz.olszowski@pum.edu.pl

**Keywords:** temporomandibular joint, temporomandibular disorders, intra-articular injections, arthrocentesis

## Abstract

**Objectives:** This overview was conducted following the Preferred Reporting Items for Overviews of Reviews guidelines and aimed to collect and compare the results of systematic reviews on temporomandibular joint injection treatment. **Methods:** Systematic reviews of randomized clinical trials on temporomandibular disorders treated with lavage or intra-articular administrations were qualified for syntheses. The final searches were conducted on 27 February 2024, without time frame restrictions. **Results:** Of the 232 identified records, 42 systematic reviews were selected. The most evidence-based conclusions call into question the clinical differences between many therapeutic approaches, including the following: (1) injectable selection for the treatment of pain and hypomobility; (2) the method of performing arthrocentesis; (3) the use of imaging when rinsing the TMJ cavity; (4) the supplementation of the extracapsular administration of unprocessed blood with intracapsular deposition in the treatment of TMJ hypermobility. **Conclusions:** Systematic reviews based solely on randomized clinical trials proved the following differences: (1) in painful temporomandibular hypomobility, a better therapeutic effect is observed with arthrocentesis followed by I-PRF administration compared to lavage alone; (2) in painful temporomandibular hypomobility, inferior- or double-compartment injection leads to better results than superior-compartment injection; (3) in temporomandibular joint recurrent dislocation, hypertonic dextrose administration is superior to placebo, although (4) unprocessed blood has a better effect than hypertonic dextrose. PROSPERO registration number: CRD42024496142.

## 1. Introduction

### 1.1. Background

Temporomandibular joints (TMJs) are paired, hidden on both sides under the skin of the preauricular area and under the branches of the facial nerve. A single TMJ consists of the mandibular fossa and the articular tubercle on the temporal bone, which form the acetabulum, and the condylar process of the mandible, which constitutes the articular head. These are separated by an articular disc, which allows for anatomical and functional distinction. All the described structures are surrounded by a joint capsule filled with synovial fluid. Ligaments and muscles provide stability to the above-described structures, as well as causing and limiting jaw movements [1].

Disfunctions of the TMJs or the muscles that move them are called temporomandibular disorders (TMDs). Their average global incident rate is 34% and they more frequently affect women [2]. Improper functioning of the articular disc leads to its displacement, which manifests itself acoustically. Gradually progressing with age, degeneration causes the thinning of articular cartilage, followed by erosive and productive changes in the bones. Stopping, and, more importantly, reversing the described processes is considered a current challenge. Complex TMDs therapy involves treatment in the field of psychology, physiotherapy, pharmacotherapy, orthodontics, dental prosthetics, and maxillofacial surgery. The latter is generally reserved for the most severe cases and ranges from injections into the joint cavity to joint replacement [3]. The least invasive of the surgical techniques are intracapsular injections.

Injection treatment of TMJs is currently one of the recognized therapeutic techniques. General indications for the use of injection techniques in the treatment of TMJs are Wilkes II-V diagnoses [4]. Due to its invasive nature, only patients who have exhausted the less invasive treatment options are typically eligible for injection treatment. Nevertheless, indications for TMJ injections may already occur in adolescence, when juvenile idiopathic arthritis is diagnosed [5].

Depending on the injectable substance used, injection treatment provides pain relief and increases the range of mandibular mobility or reduces the frequency of episodes of TMJ habitual dislocations [6,7,8]. Rinsing of the TMJs is performed as an independent procedure or precedes the injection of the active substance [9]. The most frequently used injectables include (1) hyaluronic acid, which is naturally the main component of synovial fluid, (2) autologous blood and centrifuged blood products, (3) anti-inflammatory drugs, (4) hypertonic dextrose irritant, and (5) local anesthetics [10,11,12,13,14,15].

More and more attention is being paid to aspects of injection therapy other than the type of preparation administered. Differences in the therapeutic protocols proposed by different groups of scientists are significant and include (1) the specific site of deposition (compartments of the joint cavity and pericapsular tissues); (2) the volume of the substance injected; (3) the number of interventions; (4) intervals between interventions [16,17,18,19].

In the case of rinsing the joint cavity, attempts are also made to examine the differences between the following techniques: (1) two-needle; (2) two-way needle; (3) one-way needle (pumping technique) [20,21,22,23,24]. Furthermore, arthrocentesis protocols also differ in terms of the following: (1) the type of irrigant; (2) the volume of the rinsing agent; (3) single use or repetition of the intervention at different time intervals [17,23,25]. It is common to combine lavage with active substance administration in one intervention. Finally, it is possible to mix different injectables in one dose.

### 1.2. Rationale

Combining the possibilities mentioned above allows for thousands of configurations of test and control samples. The multitude of injectable substances used, their dosing protocols, and proposals for complex treatment, including arthrocentesis, clearly demonstrate the lack of established therapeutic protocols. The same issues cause difficulties in compiling randomized controlled trials in systematic reviews. However, reviews with a lot of scientific evidence in the discussed field are already being created and provide invaluable assistance to clinicians making therapeutic decisions.

### 1.3. Objectives

This overview of systematic reviews was conducted to summarize the highest quality evidence in the field of temporomandibular joint injections and lavage.

## 2. Methods

The overview of reviews was carried out following the “Preferred Reporting Items for Overviews of Reviews (PRIOR), a protocol for the development of a reporting guideline for overviews of reviews of healthcare interventions” [26]. The Prospective Register of Systematic Reviews (PROSPERO) registered the review protocol under number CRD42024496142.

### 2.1. Eligibility Criteria

The eligibility criteria are detailed in Table 1.

### 2.2. Information Sources

Medical databases were searched using the Association for Computing Machinery: Guide to Computing Literature, Bielefeld Academic Search Engine, Google Scholar, and National Library of Medicine: PubMed engines [27,28,29,30]. The main searches were conducted on 27 July 2023, and updated searches on 27 February 2024.

### 2.3. Search Strategy

The following search strategy was applied:

Temporomandibular AND (injection OR injections OR intraarticular OR intra-articular OR intracavitary OR intra-cavitary OR periarticular OR peri-articular OR arthrocentesis OR lavage OR rinse OR rinsing) AND systematic AND review.

Detailed queries adapted to the specificity of search engines are presented in Table A1.

### 2.4. Selection Process

The results were automatically limited using the filters available in search engines (Table A1). Records were then entered into Rayyan’s automation tool (Qatar Computing Research Institute, Doha, Qatar and Rayyan Systems, Cambridge, MA, USA), which identified potential duplicates [31]. Records were manually deduplicated and blindly screened based on the content of the titles and abstracts (K.C. and M.C.). In cases of the unanimous acceptance of assessments, the given record was promoted to the next stage. The final selection was made based on full-text evaluation (N.T., M.C., and, if necessary, K.C. with a casting vote). The overlapping time frame did not disqualify systematic reviews from further proceedings.

### 2.5. Data Collection Process

Data were independently extracted by two authors (N.T. and K.L.) without using automation tools. In case of discrepancies, they were discussed, and any remaining disagreements were resolved by a third researcher (M.C.).

### 2.6. Data Items

Items necessary to identify the paper (first author, year of publication), characteristics of systematic reviews (coverage dates, number of primary studies and their participants, diagnoses, type of interventions in the study, and control groups), and outcomes (ranges of changes in pain intensity, mandibular abduction, quality of life indices, and qualitative conclusions) were collected. The diagnoses in the areas of internal derangement and isolated osteoarthritis were collectively named TMJ hypomobility to distinguish them from recurrent TMJ dislocation, whose treatment is supposed to have the opposite effect, i.e., limiting the range of mandibular abduction. Generalized arthritis manifesting itself, among others, in the temporomandibular joint area was discussed separately. Data were extracted from the bodies of the reviews and not from the content of the source studies.

### 2.7. Risk of Bias Assessment

The risk of bias in systematic reviews was assessed using the ROBIS tool (K.L. and F.B., and, if necessary, M.C. with a casting vote) [32]. ROBIS helps determine the risk of bias in systematic reviews when preparing guidelines and overviews. Assessments were made of the eligibility criteria of the studies, the identification and selection of studies, data collection and the evaluation of studies, synthesis, and the conclusions. [32]

### 2.8. Synthesis Methods

A matrix of comparisons between individual interventions was presented graphically to illustrate the conclusions supported by randomized controlled trials and areas for further research.

## 3. Results

### 3.1. Systematic Review Selection

**Figure 1:**
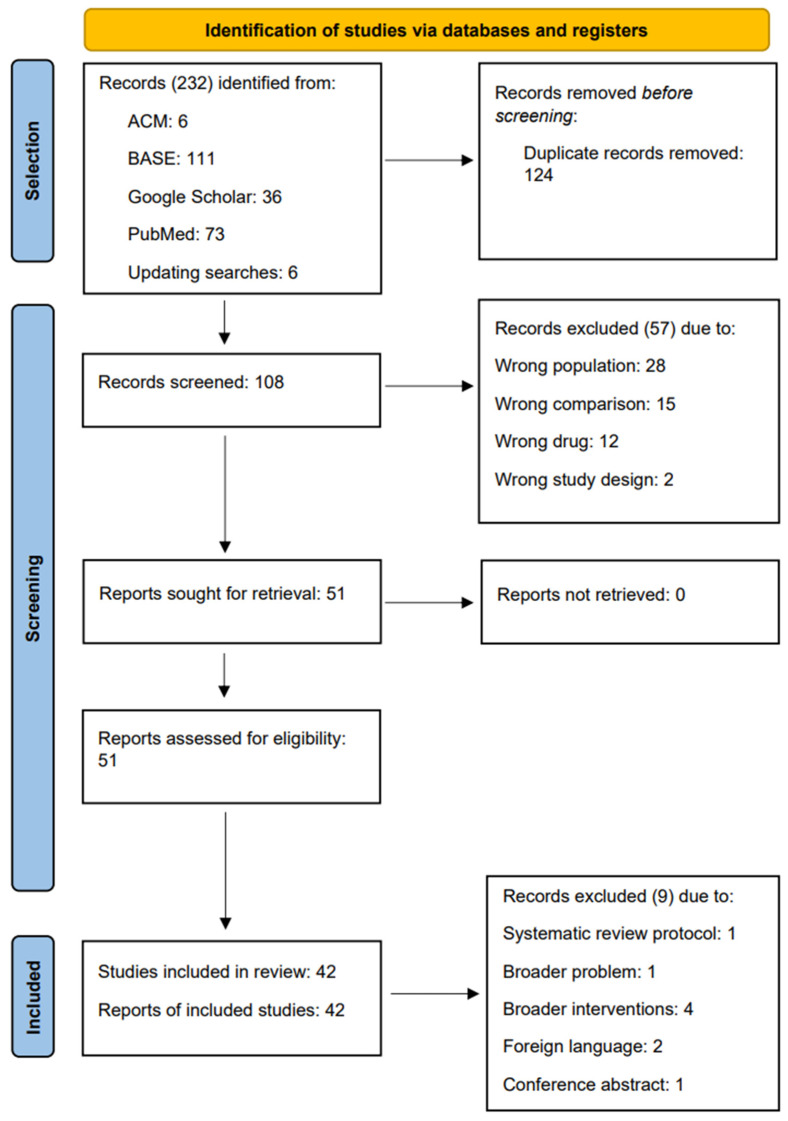
Flow diagram.

The search with four engines led to the identification of 232 records (Table A2), of which 124 were rejected in the deduplication process. The remaining 108 abstracts were screened, and 51 were qualified for full-text evaluation. Ultimately, 42 studies were included in the review. The selection process is presented in Figure 1.

### 3.2. Characteristics of Systematic Reviews

Table 2 summarized all thematically consistent systematic reviews. Reviews based solely on randomized controlled trials were promoted to risk of bias assessment and syntheses.

### 3.3. Primary Study Overlap

**Figure 2:**
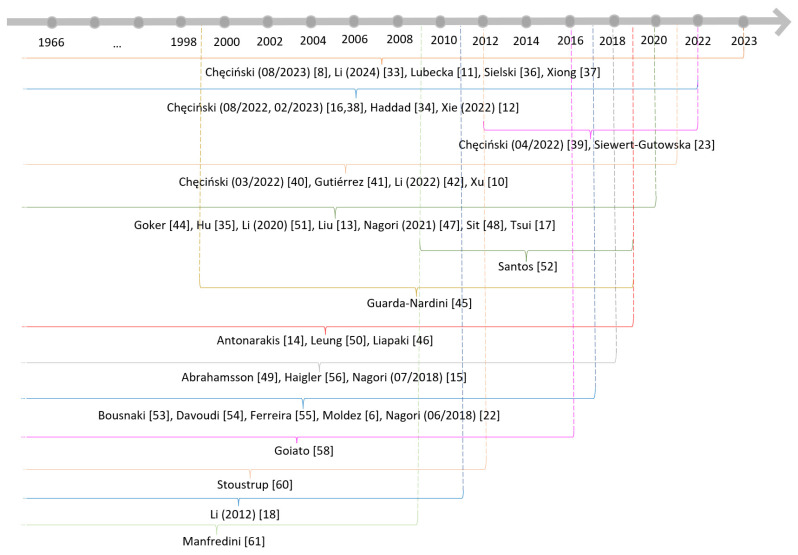
Overlap of primary studies in systematic reviews.

The overlap in time frames for systematic reviews is presented in Figure 2.

### 3.4. Risk of Bias in Systematic Reviews

The risk of bias was assessed for systematic reviews based on randomized controlled trials and is presented in Table 3. Reviews whose risk of bias was assessed as low were included in the syntheses.

### 3.5. Synthesis of Results

#### 3.5.1. Generalized Osteoarthritis

According to Xiong et al., PRP injection therapy can safely and effectively improve functionality in patients suffering from osteoarthritis [37]. It can produce positive analgesic outcomes in patients with osteoarthritis of the knee, TMJ, and ankle. However, PRP injection therapy did not significantly reduce pain in patients with hip osteoarthritis. Moreover, Leukocyte-Poor Platelet-Rich Plasma (LP-PRP) had a better analgesic outcome than Leukocyte-Rich Platelet-Rich Plasma (LR-PRP) [37].

#### 3.5.2. TMJ Hypomobility

Table 4 and Table 5 summarize the outcomes of qualified systematic reviews on different methods of TMJ hypomobility treatment. Each approach, presented in the first row, is compared to the other methods (in the first column). The completed cells correspond to comparisons that were subject to systematic reviews. The results of these reports are briefly presented. Empty cells represent comparisons that have not yet been subjected to synthetic secondary research.

#### 3.5.3. Recurrent TMJ Dislocation

Table 6 concerns TMJ recurrent dislocation, also known as TMJ hypermobility. Despite the similar nomenclature, the diagnosis of the latter represents the opposite of hypomobility of the temporomandibular joint. Therefore, there is a separate comparison matrix of the treatment methods, which also differ. In recurrent TMJ dislocation, the treatment is aimed not only at eliminating the pain but also at reducing the mandibular abduction instead of increasing it.

## 4. Discussion

### 4.1. Main Findings

Only HA versus placebo for TMJ hypomobility and HD versus placebo for TMJ hypermobility comparisons were assessed in two systematic reviews each.

Moldez et al. identified three studies (out of the seven included in their systematic review) supporting the statement that the intra-articular administration of HA leads to better results in the treatment of TMJ hypomobility and reduces the associated pain more than a placebo [6]. Xie et al. included nine randomized control trials supporting the opposite statement, that the intra-articular administration of HA does not improve pain or maximum mouth-opening compared to placebo administration [12]. Both systematic reviews were rated as having a low risk of bias in this overview. The inconsistent results of the two independent systematic reviews encourage further primary research. Attention should also be paid to the details of the HA administration protocol, such as the presence of a preceding lavage, the number of injections, and the length of the intervals between interventions. In addition, the type and amount of HA preparation are worth considering. These issues are already being raised in individual clinical studies, but the paucity of material makes it challenging to undertake appropriate syntheses.

Both Sit et al. and Nagori et al. proved that intra-articularly administered HD was significantly more effective than placebo in TMJ recurrent dislocation [15,48]. The report by Sit et al. was based on ten randomized control trials [48]. Nagori et al. qualified three studies with the same level of evidence [15]. Both systematic reviews were rated as low-risk. This suggests consensus among researchers that intra-articular HD injections resolve pain and reduce TMJ hypermobility better than placebo. The time frames of the discussed reviews overlap, which cannot be underestimated.

### 4.2. General Interpretation of the Results

#### 4.2.1. AC in TMJ Hypomobility

AC with a small volume of lavage fluid (less than 150 mL) is as effective, if not more, as a high-volume procedure [17]. A USG-guided AC does not lead to a better outcome than an unguided intervention [50]. Combining AC with the administration of CS does not provide any superior effect compared to AC alone [54]. Sole TMJ rinsing was proved to have an inferior effect compared to AC followed by I-PRF administration [36]. Combining AC with HA led to similar effects to combining it with I-PRF [62].

#### 4.2.2. CS in TMJ Hypomobility

The effect of the intra-articular administration of CS in TMJ hypomobility cases does not differ from that of the placebo control. It does not provide an advantage in terms of pain relief and TMJ mobility [12]. Another systematic review revealed no significant differences between the intra-articular administration of CS and HA [6].

#### 4.2.3. HA in TMJ Hypomobility

The advantage of using HA compared to a placebo is questionable. It was demonstrated in one systematic review and denied in another [6,12]. Moreover, HA administration also showed no significant difference compared to CS [6].

#### 4.2.4. PRP in TMJ Hypomobility

The intra-articular administration of PRP was proved to provide no better outcomes than using a placebo [12].

#### 4.2.5. Local Anesthetics in TMJ Hypomobility

The advantage of using bupivacaine over placebo was observed in the first 24 h of follow-up only [11]. In more extended observations, local anesthetics do not provide a better effect than a placebo.

#### 4.2.6. Different Compartment Injections in TMJ Hypomobility

Injection into the superior compartment only was less efficient than inferior- or double-compartment injections. This conclusion is also supported by a more recent systematic review based on primary studies with varying levels of evidence [16,18].

### 4.3. Limitations of the Evidence

A significant number of eligible systematic reviews were based on studies other than randomized controlled trials and were thus omitted from the syntheses [14,23,38,39,40,42,44,47,52,56,58,59,61,63]. Some systematic reviews based only on randomized controlled trials had a high or unclear risk of bias, which further decreased the number of synthesized systematic reviews [13,22,34,35,41,43,45,46,49,53,55,57,60].

### 4.4. Limitations of the Overview Methods

Only papers in English were included.

### 4.5. Strengths of the Overview

The strengths of this overview are the unlimited time frame, the low risk of bias in the assessment of source reports, and the conclusions, which were based solely on systematic reviews of randomized clinical trials.

### 4.6. Implications

The identified evidence suggests that some procedures can be simplified without loss to the patient, which may make treatment easier, faster, and cheaper. The use of low-volume, non-imaging arthrocentesis for painful limitations of mandibular mobility and exclusively using the extracapsular deposition of autologous unprocessed blood for habitual TMJ luxation should be considered. On the other hand, some additional measures were proven to be valid. In cases of limited jaw mobility, the administration of I-PRF after arthrocentesis and injections into the less accessible lower TMJ compartment seem to be justified.

## 5. Conclusions

In painful temporomandibular hypomobility, a better therapeutic effect is observed with injectable platelet-rich fibrin administration preceded by arthrocentesis than when using arthrocentesis alone. For the same diagnosis, inferior- or double-compartment injection leads to better results than superior-compartment deposition. In temporomandibular joint recurrent dislocation, hypertonic dextrose administration is superior to placebo but inferior to unprocessed autologous blood.

## Figures and Tables

**Table 1:** Eligibility criteria.

	Inclusion Criteria	Exclusion Criteria
Problem	Temporomandibular disorders	Cadaver or animal studies
Intervention	Arthrocentesis and/or intra- or peri-articular injection	More invasive cointervention, e.g., arthroscopy (non-surgical treatment, e.g., physiotherapy, pharmacotherapy, and/or splint therapy was allowed)
Control (applies to the synthesis of systematic reviews on randomized controlled trials)	Other eligible interventions or placebo administration	No randomization
Outcomes (applies to the synthesis of systematic reviews on randomized controlled trials)	Articular pain, mandibular abduction, or quality of life index	No quantitative assessment
Timeframe	Unlimited	Unaccepted paper, e.g., in the preprint stage
Settings	Systematic reviews published in English	No eligibility criteria, information sources, or search strategy

**Table 2:** Included systematic reviews.

First Author, Publication Year	Coverage	Source Studies	Total Number of Patients	Diagnosis	Unified Diagnosis	Intervention or Administered Substance	Control in Randomized Controlled Trials
Li, 2024 [33]	Until 2023	7 randomized controlled trials	243	TMJ internal derangement	Generalized osteoarthritis, TMJ hypomobility	PRP	HA
Chęciński, 08/2023 [8]	Until 2023	7 randomized controlled trials	390	TMJ hypermobility	TMJ hypermobility	UB	HD or placebo
Chęciński, 02/2023 [16]	Until 2022	4 studies with varying evidence	337	Various	TMJ hypomobility	Inferior TMJ space injection	N/A
Haddad, 2023 [34]	Until 2022	7 randomized controlled trials	359	Various	Generalized osteoarthritis	AC + PRP	AC + saline or AC + HA
Hu, 2023 [35]	Until 2020	4 randomized controlled trials	144	TMDs	TMJ hypomobility	Ultrasound-guided AC	Conventional AC
Lubecka, 2023 [11]	Until 2023	8 randomized controlled trials	252	TMJ arthralgia and hypomobility	TMJ hypomobility	Articaine, bupivacaine, lidocaine, mepivacaine	SS/morphine/HA/HD
Sielski, 2023 [36]	Until 2023	8 randomized controlled trials	213	TMDs	Generalized osteoarthritis	I-PRF	HA/SS injection or AC
Siewert-Gutowska, 2023 [23]	2012–2022	25 studies with varying evidence	N/S (1099 interventions)	Disc displacement with or without reduction	TMJ hypomobility	AC	N/A
Xiong, 2023 [37]	Until 2023	24 randomized controlled trials	1344	Knee/hip/ankle/TMJ OA	Generalized osteoarthritis	PRP	HA/SS
Xu, 2023 [10]	Until 2021	12 randomized controlled trials	421	TMDs	Generalized osteoarthritis	HA, PRP, PRF	RL/SS
Chęciński, 08/2022 [38]	Until 2022	5 studies with varying evidence	51	TMDs	TMJ hypomobility	AC + MSCs or MSCs	N/A
Chęciński, 04/2022 [39]	2012–2022	52 studies with varying evidence	N/S	TMDs	TMJ hypomobility or recurrent TMJ dislocation	Various	N/A
Chęciński, 03/2022 [40]	Until 2021	16 studies with varying evidence	1007	TMJ arthralgia	TMJ hypomobility	HA	N/A
Gutiérrez, 2022 [41]	Until 2021	8 randomized controlled trials	404	Various	Generalized osteoarthritis	PRP or PGRF + AC	Saline or RL + AC or AC without injection
Li, 2022 [42]	Until 2021	26 studies with varying evidence	N/S	Disc displacement with or without reduction	TMJ hypomobility	Various	N/A
Tsui, 2022 [17]	Until 2020	16 randomized controlled trials	677	TMDs	TMJ hypomobility	AC	AC
Xie, 2022 [12]	Until 2022	9 randomized controlled trials	316	TMJ osteoarthritis	TMJ hypomobility	CS, HA, PRP	Placebo
Derwich, 2021 [43]	N/S	16 randomized controlled trials	N/S	TMJ OA	TMJ hypomobility	Various	Various
Goker, 2021 [44]	Until 2020	29 studies with varying evidence	N/S	TMDs	TMJ hypomobility	HA or AC + HA	N/A
Guarda-Nardini, 2021 [45]	1999–2019	30 randomized controlled trials	N/S	TMDs (OA, ADDwoR, ADDwR, TMJ arthralgia)	TMJ hypomobility	AC	Various
Liapaki, 2021 [46]	Until 2019	9 randomized controlled trials	434	TMJ osteoarthritis	TMJ hypomobility	HA, CS, or blood products	Various
Liu, 2021 [13]	Until 2020	9 randomized controlled trials	251	TMDs treated with arthrocenthesis	TMJ hypomobility	AC + NSAIDs or AC + opioids	None, SS, RL or HA
Nagori, 2021 [47]	Until 2020	13 studies with varying evidence	715	TMDs (OA, ADDwR, ADDwoR, TMJ inflammatory and generative diseases, TMJ pain/clicking, restricted MMO)	TMJ hypomobility	Single-puncture AC	N/A
Sit, 2021 [48]	Until 2020	10 randomized controlled trials	336	TMDs diagnosed by any pre-defined or specified diagnostic criteria	Recurrent TMJ dislocation, TMJ hypomobility	HD	Various
Abrahamsson, 2020 [49]	Until 2018	8 randomized controlled trials	338	TMJ luxation	Recurrent TMJ dislocation	ABI or HD	Placebo or IMF
Antonarakis, 2020 [14]	Until 2019	11 studies with varying evidence	334	Juvenile idiopathic arthritis with TMJ involvement	Generalized osteoarthritis	CS	N/A
Leung, 2020 [50]	Until 2019	4 randomized controlled trials	144	Internal derangement of TMJ	TMJ hypomobility	Ultrasound-guided AC	Conventional AC
Li, 2020 [51]	Until 2020	11 studies with varying evidence	442	TMDs; Pain due to arthrogenic, with or without myogenic TMDs	TMJ hypomobility	AC	N/A
Santos, 2020 [52]	2009–2019	7 studies with varying evidence	N/S (over 313)	TMDs (including OA of the TMJ)	TMJ hypomobility	HA	N/A
Bousnaki, 2018 [53]	Until 2017	6 randomized controlled trials	323	degenerative TMDs (including TMJ-OA, disc displacement with osteoarthritic lesions)	TMJ hypomobility	PRP	HA, SS, or RL
Davoudi, 2018 [54]	Until 2017	7 randomized controlled trials	397	Any kind of TMDs (arthralgia, osteoartheria, osteoarthritis, juvenile idiopathic arthritis, internal derangement)	Generalized osteoarthritis and TMJ hypomobility	AC + CS	HA, SS, or RL
Ferreira, 2018 [55]	Until 2017	21 randomized controlled trials	882	Osteoarthritis, anterior displacement of the TMJ disc with or without reduction, internal derangement of the TMJ	TMJ hypomobility	HA	Various
Haigler, 2018 [56]	Until 2018	5 studies with varying evidence	285	TMJ OA	TMJ hypomobility	PRP or PRGF	N/A
Moldez, 2018 [6]	Until 2017	7 randomized controlled trials	425	OA and/or internal derange- ment of the TMJ	Generalized osteoarthritis and TMJ hypomobility	CS or NaH	Placebo
Nagori, 07/2018 [15]	Until 2018	3 randomized controlled trials	75	Painful TMJ hypermobility (subluxation or dislocation)	Recurrent TMJ dislocation	HD	SS
Nagori, 06/2018 [22]	Until 2017	5 randomized controlled trials	210	TMDs (OA/ADDwoR/ADDwR)	TMJ hypomobility	Single-puncture AC	Double needle AC
Iturriaga, 2017 [57]	N/S	2 randomized controlled trials	87	TMJ OA	TMJ hypomobility	HA	SS or RL
Goiato, 2016 [58]	Until 2016	8 studies with varying evidence	350	TMDs (OA/inflammatory joint disorder/rheumathoid arthritis)	Generalized osteoarthritis	AC + HA	N/A
Varedi, 2015 [59]	N/S	7 studies with varying evidence	122	Chronic recurrent TMJ dislocation	Recurrent TMJ dislocation	UB	N/A
Stoustrup, 2013 [60]	Until 2012	7 studies with varying evidence	268	TMJ arthritis in juvenile idiopathic arthritis (JIA)	Generalized osteoarthritis	CS	N/A
Li, 2012 [18]	Until 2011	4 randomized controlled trials	349	TMDs diagnosed by clinical and/or radiological assessment	Generalized osteoarthritis	Inferior or double TMJ spaces injection	Superior TMJ space injection
Manfredini, 2010 [61]	Until 2009	19 studies with varying evidence	604	TMDs (TMJ disk displacement, inflammatory degenerative disorders)	TMJ hypomobility	HA	N/A

ABI—autologous blood injection; AC—arthrocentesis; ADDwoR—anterior disc displacement without reduction; ADDwR—anterior disc displacement with reduction; CS—corticosteroids; HA—hyaluronic acid; HD—hypertonic dextrose; IMF—intermaxillary fixation; I-PRF—Injectable Platelet-Rich Fibrin; MMO—maximum mouth opening; MSCs—mesenchymal stem cells; N/A—not applicable; N/S—not specified; NSAIDs—non-steroidal anti-inflammatory drugs; OA—osteoarthritis; PRGF—platelet-rich growth factor; PRP—platelet-rich plasma; RL—Ringers’ lactate; SS—saline solution; TMDs—temporomandibular disorders; TMJ—temporomandibular joint; UB—unprocessed blood.

**Table 3:** Risk of bias in systematic reviews.

First Author, Publication Year	Study Eligibility Criteria	Identification and Selection of Studies	Data Collection and Study Appraisal	Synthesis and Findings	Risk of Bias in the Review
Li, 2024 [33]	Low	Low	Low	Low	Low
Chęciński, 08/2023 [8]	Low	Low	Low	Low	Low
Haddad, 2023 [34]	Low	Low	High	Low	High
Hu, 2023 [35]	Low	Low	High	Low	High
Lubecka, 2023 [11]	Low	Low	Low	Low	Low
Sielski, 2023 [36]	Low	Low	Low	Low	Low
Xiong, 2023 [37]	Low	Low	Low	Low	Low
Xu, 2023	Low	Low	High	Low	Low
Gutiérrez, 2022 [41]	Low	Low	High	High	High
Tsui, 2022 [17]	Low	Low	Low	Low	Low
Xie, 2022 [12]	Low	Low	Low	Low	Low
Derwich, 2021 [43]	Low	Low	Low	Unclear	Unclear
Guarda-Nardini, 2021 [45]	Low	Low	Unclear	Unclear	Unclear
Liapaki, 2021 [46]	Low	Low	Unclear	High	High
Liu, 2021 [13]	Low	Low	Unclear	Unclear	Unclear
Sit, 2021 [48]	Low	Low	Low	Low	Low
Abrahamsson, 2020 [49]	Low	Low	High	Low	High
Leung, 2020 [50]	Low	Low	Low	Low	Low
Bousnaki, 2018 [53]	Low	Low	High	Low	High
Davoudi, 2018 [54]	Low	Low	Low	Low	Low
Ferreira, 2018 [55]	Low	Low	High	Low	High
Moldez, 2018 [6]	Low	Low	Low	Low	Low
Nagori, 07/2018 [15]	Low	Low	Low	Low	Low
Nagori, 06/2018 [22]	Low	Low	High	Low	High
Iturriaga, 2017 [57]	Low	Low	High	Low	High
Stoustrup, 2013 [60]	Low	High	High	Unclear	High
Li, 2012 [18]	Low	Low	Low	Low	Low

**Table 4:** Comparison matrix of systematic review conclusions regarding injected substances in TMJ hypomobility.

	Placebo	AC	AC + CS	CS	AC + HA	HA	AC + I-PRF	PRP	Bupivacaine
**Compared to placebo**	X			No better effect (Xie 2022 [12])		Better (Moldez 2018 [6]); No better effect (Xie 2022 [12])		No better effect (Xie 2022 [12])	Better up to 24 h (Lubecka 2023 [11])
**Compared to AC**		X	No significant difference (Davoudi 2018 [54])				Better effect (Sielski 2023 [36])		
**Compared to AC + CS**		No significant diffference (Davoudi 2018 [54])	X						
**Compared to CS**	Same effect (Xie 2022 [12])			X		No statistically significant difference (Moldez 2018 [6])			
**Compared to AC + HA**					X		May lead to comparable clinical outcomes (Li 2024 [33])		
**Compared to HA**	Worse (Moldez 2018 [6]); Same effect (Xie 2022 [12])			No statistically significant difference (Moldez 2018 [6])		X			
**Compared to AC + I-PRF**		Worse effect (Sielski 2023 [36])			May lead to comparable clinical outcomes (Li 2024 [33])		X		
**Compared to PRP**	Same effect (Xie 2022 [12])							X	
**Compared to** **Bupicavaine**	No better effect (Lubecka 2023 [11])								X

AC—arthrocentesis; CS—corticosteroids; HA—hyaluronic acid; I-PRF—Injectable Platelet-Rich Fibrin; PRP—platelet-rich plasma.

**Table 5:** Comparison matrix of systematic review conclusions regarding injection techniques in TMJ hypomobility.

	AC	Small- Volume (<150 mL) AC	Ultrasound Guided AC	Inferior- or Double-Compartment Injection	Superior-Compartment Injection
**Compared to AC**	X	At least as effective, if not more (Tsui 2022 [17])	No better clinical outcomes (Leung 2020 [50])		
**Compared to small- volume (<150mL) AC**	As effective or less (Tsui 2022 [17])	X			
**Compared to ultrasound guided AC**	No better clinical outcomes (Leung 2020 [50])		X		
**Compared to inferior- or double-compartment injection**				X	Worse effect (Li 2012 [18])
**Compared to superior-compartment injection**				Better effect (Li 2012 [18])	X

AC—arthrocentesis.

**Table 6:** Comparison matrix of systematic review conclusions regarding recurrent TMJ dislocation.

	Placebo	HD	UB	Intracavitary + Pericapsular UB	Pericapsular UB
**Compared to placebo**	X	Significant reduction in mouth-opening and associated pain (Nagori 07/2018 [15]); Conferred a large positive effect that met the criteria for clinical relevance in the treatment of temporomandibular joint pain (Sit 2021 [48])			
**Compared to HD**	Did not lead to a reduction in mouth opening and associated pain (Nagori 07/2018 [15]); Worse effect (clinically relevant) in the treatment of temporomandibular joint pain (Sit 2021 [48])	X	Was more efficient in limiting temporomandibular dislocations in a 3-month observation (Chęciński 08/2023 [8])		
**Compared to UB**		Was less efficient in limiting temporomandibular dislocations in a 3-month observation (Chęciński 08/2023 [8])	X		
**Compared to intracavitary + pericapsular UB**				X	No difference was observed (Chęciński 08/2023 [8])
**Compared to pericapsular UB**				No difference was observed (Chęciński 08/2023 [8])	X

HD—hypertonic dextrose; UB—unprocessed blood.

## Data Availability

All collected data are included in the content of this article. The protocol was not published prior to the publication of this overview. PROSPERO registration number: CRD42024496142.

## Appendix A

**Table A1:** Search queries with filters applied.

Search Engine	Search Query
Association for Computing Machinery: Guide to Computing Literature	[All: temporomandibular] AND [[All: injection] OR [All: injections] OR [All: intraarticular] OR [All: intra-articular] OR [All: intracavitary] OR [All: intra-cavitary] OR [All: periarticular] OR [All: peri-articular] OR [All: arthrocentesis] OR [All: lavage] OR [All: rinse] OR [All: rinsing]] AND [All: review] AND [Title: systematic]
Bielefeld Academic Search Engine	temporomandibular AND (injection OR injections OR intraarticular OR intra-articular OR intracavitary OR intra-cavitary OR periarticular OR peri-articular OR arthrocentesis OR lavage OR rinse OR rinsing) AND review tit:systematic
Google Scholar	allintitle: temporomandibular AND (injection OR injections OR intraarticular OR intra-articular OR intracavitary OR intra-cavitary OR periarticular OR peri-articular OR arthrocentesis OR lavage OR rinse OR rinsing) AND systematic AND review
National Library of Medicine: PubMed	temporomandibular AND (injection OR injections OR intraarticular OR intra-articular OR intracavitary OR intra-cavitary OR periarticular OR peri-articular OR arthrocentesis OR lavage OR rinse OR rinsing) AND systematic [Title] AND review

**Table A2:** Identified and filtered records.

Search Engine	Identified Records	Filtered Records
Association for Computing Machinery: Guide to Computing Literature	37	6
Bielefeld Academic Search Engine	180	111
Google Scholar	about 29100	36
National Library of Medicine: PubMed	117	73
